# Statistical Characterization of the Medical Ultrasound Echo Signals

**DOI:** 10.1038/srep39379

**Published:** 2016-12-19

**Authors:** Runqiu Cai

**Affiliations:** 1 Affiliated Hospital of Nanjing University of TCM, Nanjing, China

## Abstract

Medical ultrasound echo signals provide the basic information for obtaining the ultrasonic image in medical ultrasound technology. The statistics of the ultrasound echo signals reveals the systematic structure of the medical ultrasonic image via analyzing the corresponding statistical distributions. A novel statistical distribution, the ascending order K distribution, was proposed here to model the medical ultrasound echo signals. The ascending order K distribution was developed in light of the statistical analysis of the sequential waveforms in the echo signals. Comparison with the previous statistical distributions was made to verify the superiority of the ascending order K distribution. Further discussion showed the determination of the statistical principles for the ultrasound signals can enhance our understanding of the statistical principles of the ultrasound imaging, and thus, facilitate the optimization of the ultrasound image and the tissue identification in the ultrasound diagnosis.

The statistical properties of the ultrasound echo signal have been discussed by many researchers. The early model proposed to describe the statistical properties of the ultrasound echo signal is the Rayleigh distribution^1^,^2^. This model based on the assumption that the human tissue is composed of a large number of scattering cells, both the magnitudes and the phases of the backscattered ultrasound echoes from the cells are statistically independent and obey the normal distribution^1^. The joint probability density function of these two components, representing the distribution function of the echo signal, results in a Rayleigh distribution^1^. The prerequisite for this model is the scattering properties of the cells distribute statistically uniform, the Rayleigh distribution will not hold either the number of scattering cells is not great enough to satisfy the central limit theorem or the strong individual scattering cells are presented in the scattering cross-sections^3^,^4^.

The K distribution, originally invented for radar imaging, was introduced to model the non-Rayleigh properties of the ultrasound echo signals in the absence of statistical uniformity^3^,^5^,^6^. The scattering events, occurred in the ultrasound-tissue interaction were treated as the random walk process in the K distribution^5^,^7^,^8^. The incident ultrasound pulse is scattered many times within the resolution cells, and the number of steps in the random walk is assumed to follow the negative binomial distribution^5^. The K distribution can be reduced to Rayleigh distribution in the limiting form as the number of steps tending to infinity. A more generalized form of K distribution can be attained by assigning a density function to the direction of the step, that is, the random walk process is biased^5^.

Another family of distribution function applied for describing the ultrasound echo signal is the Nakagami distribution and its generalised forms^9^,^10^,^11^. The Nakagami distribution, first proposed for modelling the attenuation of wireless signals, has the similar distribution pattern with the K distribution and the Rayleigh distribution^7^,^8^,^12^.

Evidences shown in this paper suggests that the raw ultrasound echo signal follows a novel statistical distribution rather than the previously proposed ones which are not the appropriate approaches for describing the raw ultrasound echo signal. Any statistical distribution, originally invented for describing phenomenon in other field, cannot be introduced to model the ultrasound echo signal directly without considering the theoretical details unique in ultrasound technology. The novel statistical distribution is proposed in light of the fundamental properties of ultrasound echo signals and the consideration that image processing could vary the statistical distribution of the raw ultrasound echo signals. The validity of the novel distribution is verified by analysing the statistical information from the abdominal fat tissue and the lateral lobe of liver tissue. The statistical properties and the potential application of this distribution are also discussed in this paper.

## Results

### Evaluation of the statistical distribution of ultrasound echo signals

The incident ultrasound pulses are produced by piezoelectric transducer of the ultrasound scanner with a certain centre frequency. On the effect of the incident ultrasound pulse, a single point within the scattering cross-section will oscillate sinusoidally and generate the echo signal with corresponding vibrating behaviour. As a result, the ultrasound echo signal along a vertical scanning line is sinusoidally-based at a certain moment. The sparse wave and dense wave sections of the ultrasound pulse correspond the negative and positive components of the sinusoidal waveform. The final form of the ultrasound echo signal is then determined by the characteristics of the scatters along the vertical scanning line. Considering a standard 10 MHz ultrasound scanner, the wavelength of ultrasound emitted from the scanner is around 26 μm as the average ultrasound velocity in human tissue is 1540 m/s^13^,^14^,^15^,^16^,^17^,^18^,^19^,^20^. The human tissue on the scale of 26 μm are treated as continuous scattering media here.

**Figure 1:**
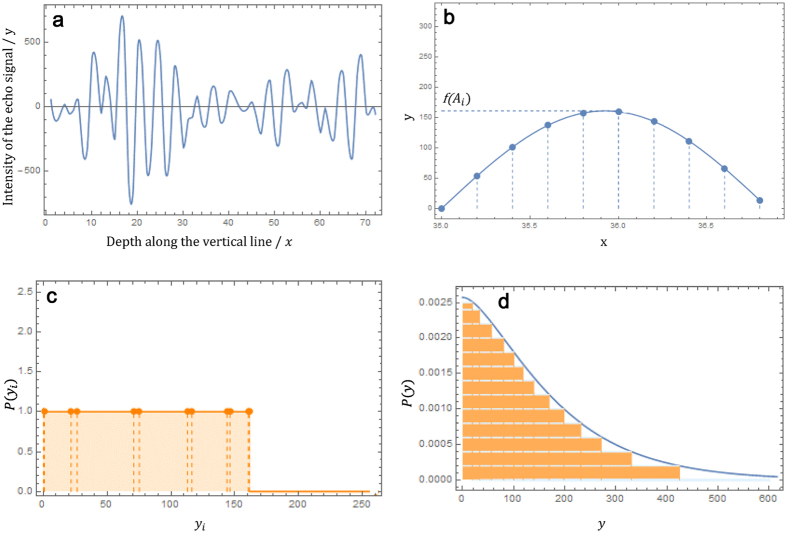
Deriving the ascending order K distribution. (**a)** the ultrasound echo signals, indicating the sinusoidal oscillating properties, along a vertical scanning line on the scale of the ultrasound wavelengths. (**b)** the point to point correspondence between the space points and the intensity of the ultrasound echo signal, namely, each ultrasound echo signal intensity occupies one space point. (**c)** the statistical representation of the point to point correspondence, the statistical distribution of one half sinusoidal period of the ultrasound echo signal can be simplified as an Heaviside function. (**d)** the statistical distribution of a given ROI section, deriving by integrating the Heaviside functions of all the half sinusoidal periods within the ROI section, can be also solved graphically as the Heaviside function piling up in the P(y)-y quadrant. The relation between *P*(*y*_*i*_) and *f*(*y*_*i*_) can be deduced from the graphical analysis.

Therefore the ultrasound echo signal takes the form of *f*(*A*)*Sin*(*x*) within each resolution cell along the vertical scanning line, where *f*(*A*) is the scattering coefficient distribution function of a given tissue structure. The amplitude of each half of the sinusoidal period is associated with the scattering coefficient distribution function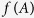.This deduction is confirmed by a close view of the ultrasound echo signal on the scale of ultrasound wavelength (Fig. 1a).

To derive the statistical distribution *P*(*y*) of the ultrasound echo signal *y*, where *y* = *Sin*(*x*), considering one half period of the sinusoidally-based waveform (Fig. 1b).By applying the concept of the set theory, every single value of *y*_*i*_ occupy a space point *x*_*i*_ along the vertical scanning line as *y*_*i*_ = *A*_*i*_*Sin*(*x*) within each half period. The probability distribution of y depends on the number of the corresponding space point *x*_*i*_ it occupies. As a result, the probability distribution of y for one half period of the waveform *A*_*i*_*Sin*(*x*) is *H*(*x*) − *H*(*x* − *A*_*i*_), where *H*(*x*) is the Heaviside step function with the amplitude of one (Fig. 1c). Re-expressing the function in the P(y) domain, *A*_*i*_ is substituted by *y*_*i*_. Then the total probability density function of y is equivalent to the summation of *H*(*y* *+y*_*i*_) − *H*(*y* − *y*_*i*_), which is 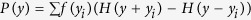
(Fig. 1d). The solution of this equation can be obtained by analysing the illustrating diagram (Fig. 1d), as the summation of *f*(*y*_*i*_) (*H*(*y* + *y*_*i*_) − *H*(*y* − *y*_*i*_)) in algebra is equivalent to piling up the Heaviside blocks *H*(*y* + *y*_*i*_) − *H*(*y* − *y*_*i*_) with the height of *f*(*y*_*i*_) graphically in the upper *P*(y) − y quadrant. Thus the relationship between *P*(y) and *f*(*y*_*i*_) can be determined as the slope of *P*(y) at point *y*_*i*_ equals to *f*(*y*_*i*_), namely 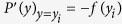, thus


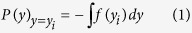


This formula suggests that the statistical distribution of ultrasound echo signal is defined by the scattering coefficient distribution. Now the K-distribution is introduced to model the scattering coefficient distribution. The reason why the K distribution is chosen for modelling the scattering coefficients is the random walk process of the K distribution, which gives rise to the various amplitude *A*_*i*_, can also be applied to describe the scattering events occurred in each half period of the sinusoidal waveform.

The n-dimensional K distribution of the scattering coefficient for each half sinusoidal period is given by^5^


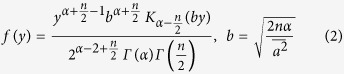


where *α* parameterizes the clustering of the scatters in the random walk process of each half sinusoidal period. 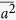 represents the deviation in the intensity of the individual scattering events and turns into parameter *σ*^2^ in Rayleigh limit.

Then the statistical distribution in two dimensional case of the ultrasound echo signal is found by the integral of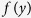.


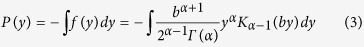


By expanding the modified Bessel functions *I*_−(*α*−1)_ and *I*_*α*−1_ into series form and evaluating the integral part^21^.


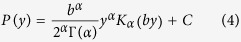


where C is an arbitrary constant.

Now evaluating the constant C and normalizing P(y), the final form of P(y) is given by (full deviation presented in Methods):


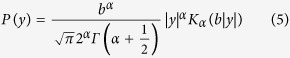


This novel statistical distribution is characterized by the ascending order of the K distribution.

### Verification of the ascending order K distribution

**Figure 2:**
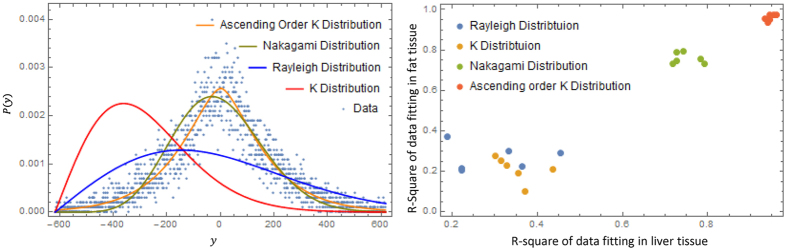
Verification of the ascending order K distribution. (**a)** an example of the data fitting, using the ultrasound echo signal from the ROI section of the abdominal fat tissue, by the ascending order K distribution and the previously proposed ones. (**b)** the regression coefficient R^2^ resulted from the data fitting, with the echo signals of both the abdominal fat tissue and the lateral lobe of liver tissue, using the ascending order K distribution and the previously proposed ones.

The ultrasound echo signal was collected from the ROI sections of the abdominal fat tissue and the lateral lobe of the liver tissue. The echo signals collection for verifying the proposed distribution was conducted using a commercially available ultrasound scanner Vinno model70 with a 10 MHz transducer. The raw ultrasound echo signals were processed in Matlab system for the statistical analysis of given ROI section. Each ROI section for certain tissue structure was selected as a 100 × 100 pixel sample box in order to provide consistent statistics. Then the statistical distributions of the sampled ultrasound echo signals were computed and were fitted by the ascending order K distribution. The data fitting process was conducted using the curve fitting toolbox in Matlab system and the curve fitting process applied the least square method^22^. Clearly, the statistical distributions of the ultrasound echo signals, precisely fitted by the ascending order K distribution, does not follow the previously proposed distributions (Fig. 2a) (Full datasets of the statistical distributions for the echo signals collected from the abdominal fat tissue and the lateral lobe of the liver are presented in Supplementary Figure 1–2).

The goodness of fit was deduced for the ascending order K distribution and previously ones. The results show that the regressing coefficients when fitted by ascending order K distribution were greater than 0.9345, by contrast, the coefficients when fitted by previously proposed distributions were all less than 0.8 especially by the Rayleigh distribution and the K distribution (Fig. 2b). As stated in the theoretical model, the ascending order K distribution, unlike the previously proposed statistical distribution, is symmetrical about the original point in the distribution function.

## Discussion

Unlike the previously proposed statistical distributions, the ascending order K distribution manifests the feature that the greatest amount of signal appears at the original point in the distribution function^1^,^5^,^7^,^8^. This is reasonable as the ultrasound echo signal with a sinusoidal waveform goes through the zero value during every half period and the varying amplitude *A*_*i*_ of the sinusoidal echo signal results the descending tendency of the statistical distribution. The K distribution tends to the Rayleigh limit as the number of clustering goes to infinity^3^,^5^, and the corresponding solution for the ascending order K distribution is given by


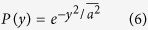


**Figure 3:**
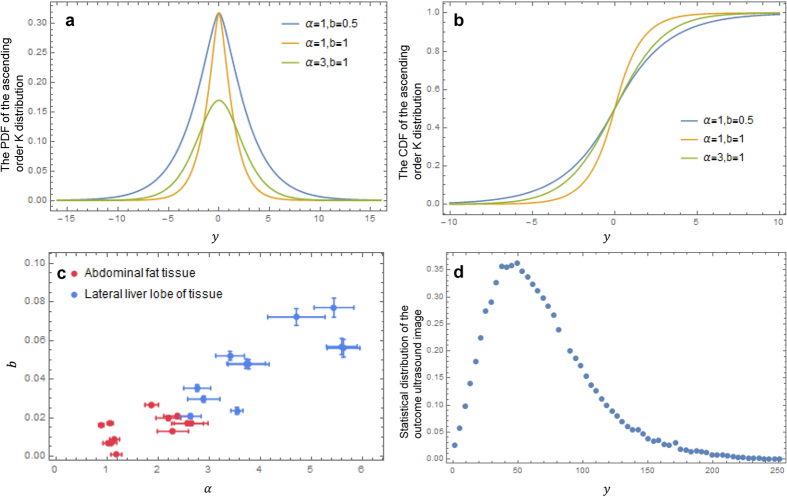
The properties of the ascending order K distribution. (**a)** the probability density function (PDF) of the ascending order K distribution. (**b)** the cumulative distribution function (CDF) of the ascending order K distribution. (**c)** the fitting results for samples from the abdominal fat tissue and the lateral lobe of liver tissue with the 95% confidential intervals of the parameters *α* and *b* shown as the error bars. **(d)** the statistical distribution of the output ultrasound image collected from the same ROI section with Fig. 2a.

This result suggests if the amount of scatters is considerably large within a certain tissue structure, then the ultrasound echo signals generated from this tissue structure approximately follow the normal distribution. The ascending order K distribution is symmetrical about the original point and monotonically decreases along the positive and the negative axis (Fig. 3a,b).

The experiments conducted in abdominal fat tissue and lateral lobe of the liver tissue have validated the ascending order K distribution to be the appropriate model for the ultrasound RF signal. The parameters of the ascending order K distribution, the clustering parameters α and the scattering fluctuation related parameter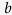, were derived in the data fitting process for all samples (Fig. 3c). The 95% confidence interval of parameter α and *b* are labelled as error bows in the diagram. The results show the clustering parameters α of the abdominal fat tissue and the lateral lobe of the liver are centralized at 1.764 and 4.015 respectively, and the parameters *b* are averaged at 0.01959 for the abdominal fat tissue and at 0.04771 for the lateral lobe of the liver tissue. Comparing the parameters suggests the statistical analysis of the raw ultrasound echo signal can provide a reliable reference for tissue typing in ultrasound diagnosis. Meanwhile, the clarification of the statistical principles of the ultrasound echo signal can assist us to enhance and optimize the ultrasound imaging algorithms.

The signal processing algorithms that turns the raw ultrasound echo signal into ultrasound image can vary the statistical distribution of the raw ultrasound echo signal. This consideration was confirmed by comparing the distributions of the raw echo signals (shown in Fig. 2a) and the output image from the same ROI section (Fig. 3d). The outcome image is collected from the terminal display where the image has been manipulated by many filtering and transform procedures.

## Methods

### Full deviation of the ascending order K distribution


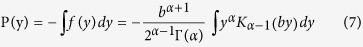


Given that


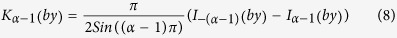


Then


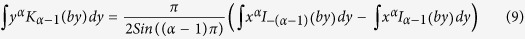


By expanding the modified Bessel function I_−(α−1)_(by) and *I*_*α*−1_(*by*) into the series


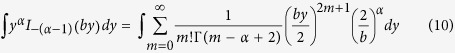



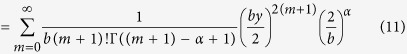



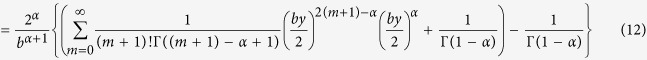



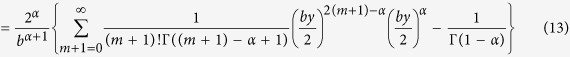



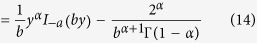


Applying the similar procedures


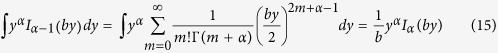


Applying Equations (14) and (15) into the Equation (9)


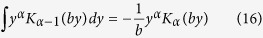


Applying Equation (16) into Equation (7) and normalising the result, the expression of P(y) is given by


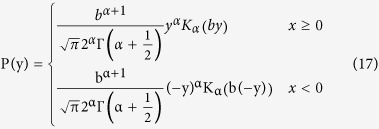



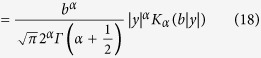


### Data Sets

The ascending order K distribution was verified in the abdominal fat tissue and the lateral lobe of the liver. The experiment for validating the ascending order K distribution was conducted using a commercially available ultrasound scanner Vinno model70 with a 10 MHz transducer. The raw ultrasound echo signals, collected from the background database of the ultrasound scanner, were decoded in Matlab system for the statistical analysis of given ROI section. To provide consistent statistics, each ROI section for certain tissue structure was selected as a 100 × 100 pixel sample box containing 10000 data points. The number of a given echo signal intensity in each ROI is counted in Matlab. Then each data sets of the ROI section were fitted by the ascending order K distribution and all data fittings have the regressing coefficients greater than 0.9345.

## Additional Information

**How to cite this article**: Cai, R. Statistical Characterization of the Medical Ultrasound Echo Signals. *Sci. Rep.* **6**, 39379; doi: 10.1038/srep39379 (2016).

**Publisher's note:** Springer Nature remains neutral with regard to jurisdictional claims in published maps and institutional affiliations.

## Supplementary Material

Supplementary Information
