# Supplementary material for: Statistical Characterization of the Medical Ultrasound Echo Signals
Source: Sci Rep. 2016 Dec 19;6:39379. doi: 10.1038/srep39379 (PMC5171708; doi:10.1038/srep39379)
Supplement: Supplementary Information [file srep39379-s1.pdf]

Statistical Characterization of the Medical Ultrasound Echo Signals

Supplementary Information

Author: Runqiu Cai

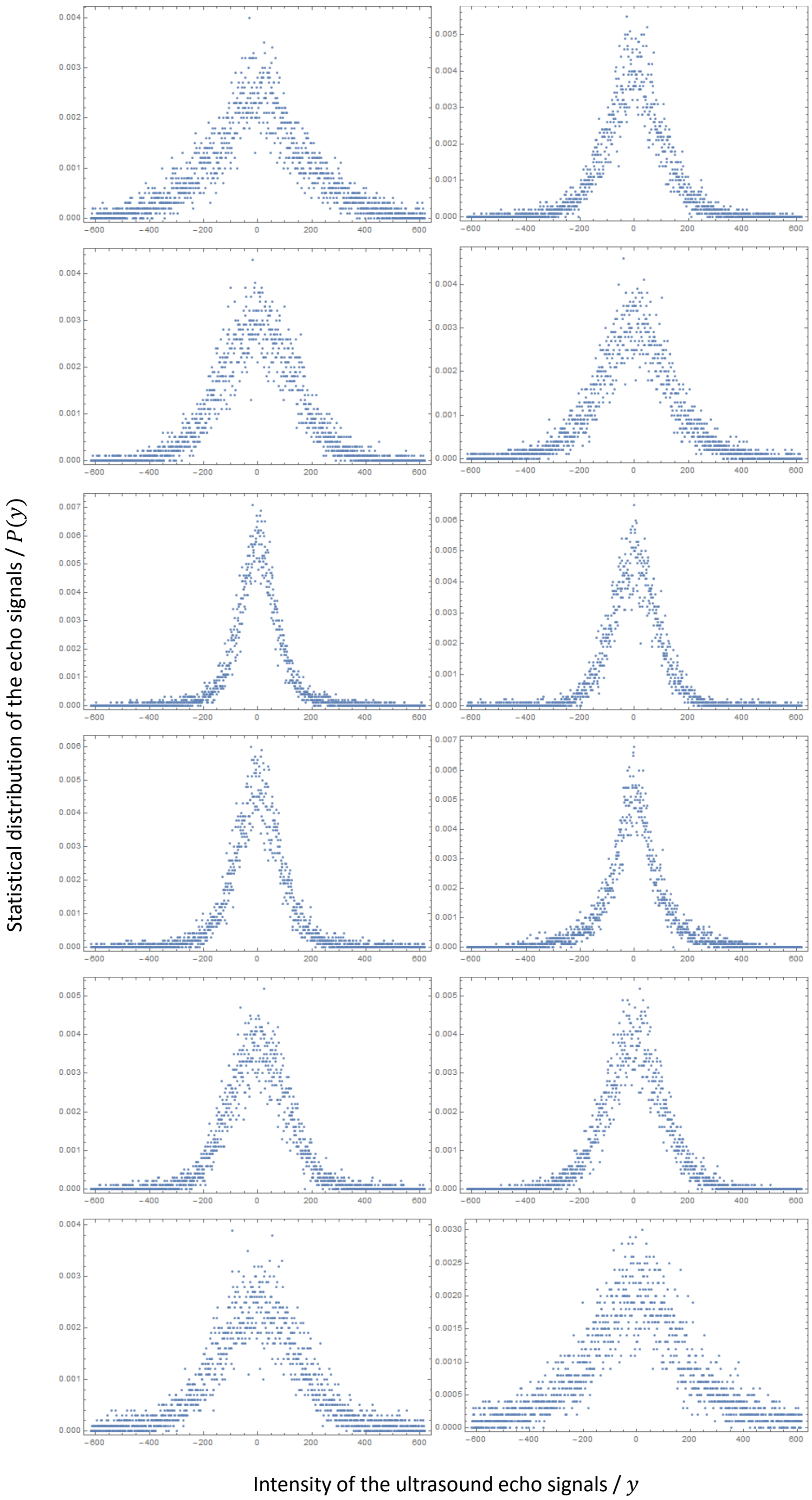

Supplementary Figure 1 | The full statistical distributions of the ultrasound echo signals collected from the ROI sections in abdominal fat tissue.

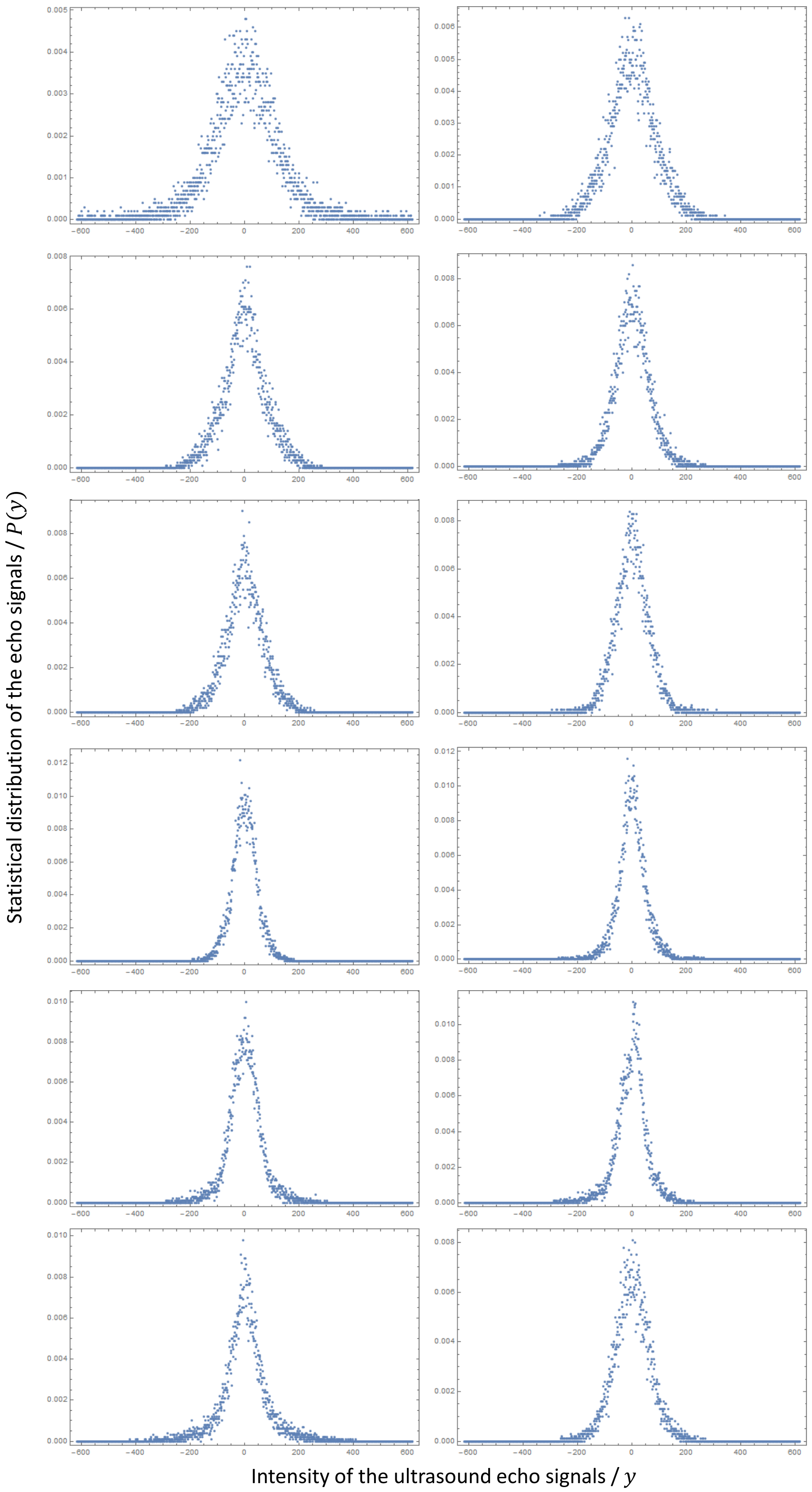

Supplementary Figure 2 | The full statistical distributions of the ultrasound echo signals collected from the ROI sections in lateral lobe of the liver.
